# Importance of Clinical and Laboratory Findings in the Diagnosis and Surgical Prognosis of Patients with Constrictive Pericarditis

**DOI:** 10.5935/abc.20170147

**Published:** 2017-11

**Authors:** Fábio Fernandes, Dirceu Thiago Pessoa de Melo, Felix José Alvarez Ramires, Ricardo Ribeiro Dias, Marcio Tonini, Vinicius dos Santos Fernandes, Carlos Eduardo Rochitte, Carlos Henrique Valente Moreira, Charles Mady

**Affiliations:** 1 Instituto do Coração (InCor) do Hospital das Clínicas da Faculdade de Medicina da Universidade de São Paulo (HCFMUSP), São Paulo, SP - Brazil; 2 Instituto de Medicina Tropical, Universidade de São Paulo, São Paulo, SP - Brazil

**Keywords:** Pericarditis, Constrictive/surgery, Diagnosis, Prognosis, Diagnostic Imaging, Pericardiectomy

## Abstract

**Background:**

International studies have reported the value of the clinical profile and laboratory findings in the diagnosis of constrictive pericarditis. However, Brazilian population data are scarce.

**Objective:**

To assess the clinical characteristics, sensitivity of imaging tests and factors related to the death of patients with constrictive pericarditis undergoing pericardiectomy.

**Methods:**

Patients with constrictive pericarditis surgically confirmed were retrospectively assessed regarding their clinical and laboratory variables. Two methods were used: transthoracic echocardiography and cardiac magnetic resonance imaging. Mortality predictors were determined by use of univariate analysis with Cox proportional hazards model and hazard ratio. All tests were two-tailed, and an alpha error ≤ 5% was considered statically significant.

**Results:**

We studied 84 patients (mean age, 44 ± 17.9 years; 67% male). Signs and symptoms of predominantly right heart failure were present with jugular venous distention, edema and ascites in 89%, 89% and 62% of the cases, respectively. Idiopathic etiology was present in 69.1%, followed by tuberculosis (21%). Despite the advanced heart failure degree, low BNP levels (median, 157 pg/mL) were found. The diagnostic sensitivities for constriction of echocardiography and magnetic resonance imaging were 53.6% and 95.9%, respectively. There were 9 deaths (10.7%), and the risk factors were: anemia, BNP and C reactive protein levels, pulmonary hypertension >55 mm Hg, and atrial fibrillation.

**Conclusions:**

Magnetic resonance imaging had better diagnostic sensitivity. Clinical, laboratory and imaging markers were associated with death.

## Introduction

Constrictive pericarditis (CP) results from loss of pericardial elasticity, which generates a restrictive syndrome. It is an infrequent cause of heart failure (HF), poorly diagnosed because of its peculiar pathophysiology.^1-3^ The inelastic pericardium prevents ventricular filling, usually leading to signs and symptoms of right HF, which can be mistaken for other causes of HF and liver diseases, hindering the patient’s outcome; because CP is a potentially curable disease, the sooner it is treated, the better the prognosis.^4,5^

Previous studies have reported the clinical profile and the value of imaging tests for diagnosing CP.^6-8^ However, data on the Brazilian population are scarce. This study was aimed at assessing the clinical characteristics, sensitivity of laboratory tests and factors related to death in a case series of patients with CP undergoing pericardiectomy in a period of 13 years at a tertiary referral center.

## Method

### Population

We studied retrospectively 95 patients diagnosed with CP and submitted to pericardiectomy from January 2002 to August 2015. Of those, 11 patients were excluded due to incomplete medical records, leaving 84 to the final analysis. 

The following clinical data were collected: age, sex, duration of symptoms, hospital length of stay, ascites, edema, pericardial knock, jugular venous distension, paradoxical pulse, Kussmaul sign, and atrial fibrillation (AF)]. Pericardial knock was characterized as a rough protodiastolic sound, coinciding with the rapid Y descent. The following laboratory tests were performed: hemoglobin, brain natriuretic peptide (BNP) and C reactive protein (CRP). Chemiluminescence immunoassay was used to measure BNP (Bayer ADVIA Centaur Bayer Diagnostics, São Paulo, SP, Brazil).

Preoperative cardiac echocardiography and magnetic resonance imaging suggested the diagnosis of CP in the presence of at least two findings compatible with constriction: pericardial thickening; septal bounce (paradoxical movement); inspiratory reduction in the mitral E wave (> 25%); and inferior vena cava dilatation. When more than one test was available, we chose the oldest.

Tuberculous pericarditis was diagnosed by use of pericardial biopsy, evidencing caseous granuloma or positive polymerase chain reaction for the bacillus. Post-radiation and post-surgical CP were defined in the presence of previous mediastinal radiotherapy and cardiac surgery, respectively. Idiopathic CP was defined when patients did not fit in any cited group.

Survival was obtained in the medical records or via telephone contact.

### Pericardiectomy

Pericardiectomy was performed via median sternotomy in all cases, without cardiopulmonary bypass. Total pericardiectomy was defined as the excision of the anterior pericardium up to the phrenic nerves and the diaphragmatic surface. The visceral and parietal pericardium was removed whenever technically possible.

### Statistical analysis

Initially descriptive analysis was performed. Measures of central trend and dispersion were presented as means and standard deviations or medians and minimum and maximum values, according to distributions. Qualitative variables were reported as absolute frequency and percentages. Chi-square or Fisher exact tests were performed to assess the differences between the groups regarding categorical variables.

All quantitative variables were tested to assess if they had a normal distribution by use of the Shapiro-Wilk test, and visually, by use of quantile-quantile graphs (Q-Q plot) or histograms. Logistic regression was used in the presence of dichotomous outcomes.

Predictors of mortality were determined by use of univariate analysis with Cox proportional hazards model. Hazard ratio (HR) and respective 95% confidence intervals (CI) and alpha values (p) were presented. Because of the small number of patients assessed in this study, multiple analysis to investigate the factors independently associated with death could not be performed.

All tests were two-tailed, an alpha error ≤ 5% was considered statically significant, and 95%CI was used in the analysis.

Data were entered in the Microsoft Excel 365 software, and analyzed in the STATA statistical program, version 13.0 (StataCorp LP, College Station, Texas, USA).

## Results

This study assessed 84 patients, with a mean age of 44 ± 17.9 years, and 77% of the male sex. On admission, all had symptoms of HF, with predominance of jugular venous distension (89%), edema (89%) and ascites (62%). The median duration of those symptoms was 24 months, ranging from 1 to 1460 days (Table 1). The mean hospital length of stay was 10 days, and at the intensive care unit, 3 days. The etiologies of CP were as follows: idiopathic (69.1%); tuberculous (21.4%); post cardiac surgery (5.9%); systemic inflammatory disease (2.4%); and post radiotherapy (1.2%).

**Table 1:** Distribution of patients with constrictive pericarditis according to clinicals and laboratory characteristics

Parameter	Value
Male sex (%)	65 (77,4%)
**Age (years)**	
mean (SD)	44.4 (± 18)
median [min - max]	44.5 [14 - 86]
Symptom duration (months)*	
median [min - max]	24 [1 -1460]
BNP (pg/dL)**	
median [min - max]	157 [14-692]
CRP (mg/dL)***	
median [min - max]	7.40 [0.72 - 142]
**Hemoglobin (g/dL)**	
median [min - max]	13 [8.0 - 17.4]
**Creatinine (mg/dL)**	
mean (dp)	1.06 (± 0.24)
**EuroScore**	
median [min - max]	0.91 [0.56 - 17.68]
Urgent surgery	23
**ICU LOS (days)**	
median [min - max]	3.00 (0 - 80)
Hospital LOS (days)	10 [0 - 91]
Atrial fibrillation (%)	29 (34.5%)
NYHA III-IV (%)	66 (78.5%)
Jugular venous distention	75 (89%)
Pericardial knock	17 (20%)
Paradoxical pulse	11 (13%)
Edema	75 (89%)
Ascites	52 (62%)
Kussmaul sign	12 (14%)
Pleural effusion	37 (44%)
Calcification on chest X-ray	19 (22%)
Lethality	9 (10.7%)

Missing data:

(*) 27;

(**) 20;

(***) 17

BNP: brain natriuretic peptide; CRP: C reactive protein; SD: standard-deviation; ICU: intensive care unit; LOS: length of stay.

Despite the advanced degree of HF, we found low levels of BNP (median, 157 [14-692] pg/mL) and high inflammatory activity (CRP: median, 7.40 [0.72-142]). On chest X-ray, pericardial calcification was identified in 19 patients (22.6%) (Table 1).

Of the echocardiographies performed, only 45 (53.6%) suggested CP, with pericardial thickening in 59 (70.2%), and respiratory variation in mitral and tricuspid flows in 38 (45.2%), indicating diastolic restriction (Table 2). Regarding cardiac resonance imaging, 73 patients underwent the exam, and 70 (95.9%) showed signs suggestive of CP. Pericardial thickening (pericardial thickness > 4 mm) was shown in 66 (90.4%) exams, the most common changes being aortocaval dilatation (70 - 95.9%) and septal bouncing (paradoxical movement of the ventricular septum, 64 - 87.6%) (Table 3).

**Table 2:** Echocardiographic variables

Echocardiographic variables	n (%)
Suggestive of CP	45 (54.2)
Non-suggestive of CP	38 (45.8)
Pericardial thickening	59 (70.2)
Respiratory variation in mitral and tricuspid flow	38 (45.2)
PASP > 55	8 (9.5)
**Ejection fraction (%)**	
median [min - max]	60 (32 - 80)
**Stroke volume (mL)**	
median [min - max]	34 (10 - 118)
**Diastolic volume (mL)**	
median [min - max]	88 (8 - 173)
**LVSD (mm)**	
median [min - max]	29 (18 - 50)
**LVDD (mm)**	
median [min - max]	45 (31 - 59)
**Left atrium (mm)**	
median [min - max]	43.5 (30 - 73)
**Aortic sinus (mm)**	
median [min - max]	30 (18 - 42)
**Ventricular septum (mm)**	
median [min - max]	8 (6 - 13)
**PW (mm)**	
median [min - max]	8 (6 - 12)

CP: constrictive pericarditis; PASP: pulmonary artery systolic pressure; LVSD: left ventricular systolic diameter; LVDD: left ventricular diastolic diameter; PW: posterior wall.

**Table 3:** Cardiac magnetic resonance imaging variables

Variables	N°	%
**Magnetic resonance imaging**		
Not performed	11	13.1
Suggestive of CP	70	95.8
Non-suggestive of CP	3	4.1
Pericardial enhancement	14	19.2
Myocardial enhancement	9	12.3
Septal bouncing	64	87.6
Aortocaval dilatation	70	95.9
Left atrial enlargement	58	79.5
**Left ventricular function (%)**		
median [min - max]	60	(25 - 82)
**Pericardial thickening (mm)**		
median [min - max]	5	(0 - 20)

CP: constrictive pericarditis.

### Survival analysis

The mean hospital length of stay was 14.6 days (standard deviation of 13.2 days) and the median hospital length of stay, 10 days [0 - 91 days]. Of the 84 patients assessed, 9 died (lethality: 10.7%), 6 (66.6%) of whom in the first 30 postoperative days (early death), and the other 3, after that period.

The most frequent cause of early death was cardiogenic shock (5 patients - 55.5%), and only one patient had mixed cardiogenic and septic shock. Regarding the three deaths occurring after the 30th postoperative day, one was preceded by mixed cardiogenic and septic shock, and the other two, by cardiogenic shock.

Early death showed no association with any of the variables studied (Table 4).

**Table 4:** Descriptive statistics of quantitative variables of patients with constrictive pericarditis according to the occurrence of death after pericardiectomy

Variable	DEATH		p^+^
	NO (n = 75)	YES (n = 9)	
	median [min - max]	median [min - max]	
Age	43 [14 - 86]	48 [23 - 69]	0.84
Symptom duration (days)	24 [2 - 1460]	12 [1 - 96]	0.485
Postop. hospital LOS	10 [3 - 91]	6 [0 - 40]	0.3
ICU LOS	3 [2 - 80]	2.5 [0 - 8]	0.464
Preop. hemoglobin	13.3 [8 - 17.4]	11.9 [9.6 - 13]	0.017
Creatinine	1.06 [0.62 - 1.75]	1.0 [0.77 - 1.58]	0.815
C reactive protein	5.54 [0.72 - 142]	32.2 [5.76 - 83.8]	0.022
BNP	143 [14 - 468]	432 [160 - 692]	0.001
EuroScore II	0.88 [0.56 - 17.68]	1.67 [0.79 - 6.0]	0.214
Ejection fraction	0.60 [0.32 - 0.80]	0.60 [0.48 - 0.70]	0.581
Stroke volume	35 [10 - 118]	32 [20 - 35]	0.691
Diastolic volume	88 [8 - 173]	83.5 [51 - 108]	0.444
LVSD	29 [18 - 50]	29 [24 - 32]	0.449
LVDD	45 [31 - 59]	44 [35 - 48]	0.401
PW	8 [6 - 12]	8 [7 - 11]	0.912
Septum	8 [6 - 13]	8 [7 - 13]	0.482
LA	44.5 [30 - 73]	42 [41 - 65]	0.409
Aortic sinus	30 [18 - 42]	31 [27 - 37]	0.293

(+) Logistic regression. Postop: postoperative; LOS: length of stay; ICU: intensive care unit; Preop: preoperative; BNP: brain natriuretic peptide; LVSD: left ventricular systolic diameter; LVDD: left ventricular diastolic diameter; PW: posterior wall; LA: left atrial diameter.

Death showed a statistically significant association with: AF; laboratory markers (preoperative hemoglobin, CRP and BNP); and echocardiographic marker (pulmonary hypertension > 55 mm Hg). The other variables in this sample showed no statistically significant association with death in CP (Tables 4 and 5).

**Table 5:** Deaths of patients diagnosed with constrictive pericarditis according to demographic, clinical and laboratory characteristics.

Variables	Total	deaths (lethality)	HR	95%CI	p^§^
Early death*					
yes	-	6 (66.6%)	-	-	
**Sex**					**0.98**
male	65	7 (10.7%)			
female	19	2 (10.5%)	0.98	0.20 - 4.75	
**Age (years)**					**0.484**
< 44	41	3 (7.13%)			
≥ 44	43	6 (13.9%)	1.93	0.48 - 7.75	
**Etiology**					**0.626**
Idiopathic	58	7(12.06%)			
Tuberculosis	18	1 (5.55%)			
Post-cardiac surgery	5	1 (20%)			
Radiotherapy	1	0			
Systemic disease	2	0			
**Atrial fibrillation**					**0.05**
yes	29	6 (20.6%)	3.87	1.01 - 16.23	
no	55	3 (5.4%)			
**NYHA ≥ 2**					**1.00**
no	16	2 (12.5%)			
yes	68	7 (10.29%)	0.93	0.19 - 4.51	
**Total symptom duration (days)^†^**					**0.356**
< 24	28	5 (17.8%)			
≥ 24	29	4 (13.7%)	0.51	0.12 - 2.13	
**ICU LOS ≥ 3 days**^‡^					0.454
no	43	5 (11.6%)			
yes	28	3 (10.7%)	0.91	0.70 - 1.16	
**Postop. hospital LOS ≥ 10 days**					**0.503**
no	37	5 (13.5%)			
yes	47	4 (10.8%)	0.54	0.14 - 2.05	
**PASP > 55 mm Hg**					**0.046**
no	60	5 (8.3%)			
yes	8	3 (37.5%)	4.5	1.38 - 24.2	
**Preop. hemoglobin**					**0.003**
< 13	33	8 (24.2%)			
≥ 13	33	0	0	-	
**C reactive protein**					**0.0**
< 7.4	26	1 (3.8%)			
≥ 7.4	26	7 (87.3%)	8.5	1.04 - 69.33	
**BNP**					**0.011**
< 157	32	0			
≥ 157	32	7 (21.8%)	NC		

CI: confidence interval; ICU: intensive care unit; LOS: length of stay; Postop: postoperative; PASP: pulmonary artery systolic pressure; Preop: preoperative; BNP: brain natriuretic peptide.

* Early death (≤ 30 postoperative days);

† 57 available data;

‡ 63 available data;

§ Fisher exact test; NC: could not be calculated.

Thus, in patients with CP, death was associated with the presence of AF, lower hemoglobin levels (< 13 g/dL), higher CRP (≥ 7.4 mg/dL) and BNP (≥ 157 pg/mL) levels, and pulmonary artery systolic pressure (PASP) > 55 mm Hg. Their respective HR are shown in Tables 5 and 6.

**Table 6:** Variables associated with death

Variable	Late death (n = 3)	Early death (n = 6)	p^+^
	median [min-max]	median [min-max]	
Age	23 [48 - 69]	23 [51 - 61]	0.884
Symptom duration (days)	24 [12 - 96]	12 [1 - 24]	0.366
Postop. hospital LOS	58 [40 - 71]	1.5 [1 - 23]	0.175
Preop. hemoglobin	9.6 [11.8 - 12.6]	12 [10.2 - 13]	0.507
BNP	683 [160 - 692]	431 [321 - 465]	0.469
Creatinine	1.1 [1.04 - 1.36]	0.95 [0.77 - 1.58]	0.48
CRP	74.5 [5.76 - 74.8]	29.4 [18.58 - 83.9]	0.491

(+) Logistic regression. Postop: postoperative; Preop: preoperative; BNP: brain natriuretic peptide; CRP: C reactive protein.

## Discussion

Despite the advances in complementary methods, in many patients, the correct diagnosis of pericardial constriction is established late, with a mean time elapsed between symptom onset and diagnosis of 24 months.

Constrictive pericarditis can simulate a large variety of clinical and cardiological syndromes, such as liver cirrhosis, myocardial failure and restrictive cardiomyopathies.^9^ The clinical findings are those of HF, with anasarca and ascites predominating over lower limb edema. Nonspecific symptoms include fatigue, anorexia, nausea, dyspepsia and weight loss. Thus, correct diagnosis requires a high index of clinical suspicion by the cardiologist.

The clinical findings reported in a case series of the Mayo Clinic were: HF (67%); chest pain (8%); abdominal symptoms (6%); restrictive symptoms (5%); atrial arrhythmias (4%); and severe liver disease (4%). In 6% of the cases, there was low cardiac output, repetitive pleural effusion and syncope. The duration of symptoms prior to surgery was also long, of 11.7 months, ranging from 3 days to 30 years.^6^

In our case series, the major symptom was biventricular NYHA functional class III-IV HF (78%), with predominance of right HF, and ascites. The patients had cardiac cachexia, increased jugular venous pulse and Kussmaul sign. Pericardial knock was an infrequent finding, observed in 20% of our cases; however, when present, it suggested CP. On auscultation, the pericardial knock is characterized by a rough, sharp, protodiastolic sound that results from the vibration of the ventricular wall during the rapid filling phase, coinciding with the rapid Y descent. It can be mistaken for the third cardiac sound, which, however, is usually lower pitched. One of the patients was being assessed for liver transplantation; however, the increase in jugular venous pulse with rapid Y descent and pericardial knock suggested the correct diagnosis of pericardial constriction. A significant number of patients (34%) had AF, probably due to the long course of their disease.

Tuberculosis, collagenosis, neoplasms and previous cardiac surgery are some etiologies of CP, which is most commonly idiopathic or secondary to viral pericarditis. In developed countries, the most frequent etiologies are post-cardiac surgery and post-radiation. Tuberculosis continues to be a frequent etiology in developing countries or in immunosuppressed patients.^1,10^

The etiologies found in our study were as follows: idiopathic (69%), tuberculosis (21%), post-cardiac surgery (6%), systemic inflammatory disease (2%), and post-radiation (1%). In the case series of the Mayo Clinic, the etiological diagnosis was established in 73% of the 135 patients studied, the major being as follows: post-cardiac surgery (18%), pericarditis (16%), and post-radiation (13%). Other less frequently reported etiologies were rheumatological diseases: rheumatoid arthritis, polymyalgia, myopericarditis, psoriatic arthritis, and Still’s disease. In that case series, the infectious causes were infrequent: one case of fungal pericarditis and two of tuberculosis.^6^

Physiological and structural data can be obtained by use of echocardiography, and cardiac tomography and resonance. Recent advances in diagnostic methods have provided complementary assessment in patients with diagnostic suspicion of constriction.^8,11-13^

Echocardiography is the first non-invasive test that can help when CP is suspected, allowing the differential diagnosis with forms of right ventricular failure, such as restrictive cardiomyopathy, pulmonary hypertension, and mitral valve disease.^14^ The major findings include pericardial thickening, better visualized on transesophageal echocardiogram, abnormal movement of the ventricular septum, dilatation and lack of inspiratory collapse of the inferior vena cava, respiratory variation in mitral (> 25%) and tricuspid (> 40%) flows, normal or increased velocity of E’ waves, and biatrial enlargement.

In our case series, only 54% of the cases had an echocardiogram suggestive of pericardial constriction. It is worth noting that 30% of the patients had no pericardial thickening, despite surgical confirmation. Thus, the diagnosis of CP and indication for pericardiectomy should not be postponed in the absence of pericardial thickening or when there is no other signal of pericardial constriction.^6,15^ It is worth noting that, in the absence of echocardiographic signs suggestive of CP, the diagnosis of CP is delayed. Many services lack more sophisticated complementary exams, such as resonance and tomography. The presence of signs and symptoms of right HF should raise the suspicion of restrictive syndrome. Regarding the topographic and etiological diagnosis of restrictive syndromes, we should consider endocardial, myocardial and, especially among us, pericardial affections. Thus, when the echocardiogram is inconclusive, other complementary exams should be performed to confirm the diagnosis of pericardial constriction, such as cardiac tomography and resonance. The later the diagnosis, the worse the prognosis.

Tomography and resonance provide a fair anatomical assessment of the pericardium and cardiac chambers. In addition, resonance imaging provides the functional assessment of diastolic restriction signs. In our study, resonance imaging suggested CP in 96% of the patients.

The pericardium has a fibrous structure with T1 and T2 hypointense characteristic as compared to the myocardium. A pericardial thickness of 2 mm is considered normal. Pericardial thickening of 4 mm suggests CP, and that of 6 mm has high specificity for the diagnosis of constriction. Approximately 10% to 20% of the patients undergoing surgery have normal pericardial thickness, which, thus, does not rule out the diagnosis. In such cases, there is visceral pericardial inflammation (epicarditis), pericardiectomy being often curative. Talreja et al.,^16^ in a retrospective study of 26 patients diagnosed with CP and normal pericardial thickness (< 2 mm), have found the following etiologies: cardiac surgery (42%), chest radiation (19%), post-myocardial infarction (12%), and idiopathic (12%).

In most cases, resonance imaging provides the presumptive diagnosis of CP. In addition to pericardial thickening, other characteristics provided by that exam, such as atypical movement of the ventricular septum and inferior vena cava dilatation, should be valued.

In only 4% of the cases, the diagnosis was complemented with catheterization and chest tomography. Thus, even with important clinical suspicion and no confirmation by complementary exams, we indicate exploratory thoracotomy, because, if the diagnosis is not performed, a poor outcome is highly likely.

Another finding in our case series was low BNP levels (median, 157 pg/mL), despite the advanced functional class HF. That could be explained by diastolic restriction and lower parietal tension, determining a lower stimulus to BNP release. In previous studies, CP had lower BNP levels as compared to those of other restrictive syndromes, such as restrictive cardiomyopathy, and that measurement could be useful for the differential diagnosis with other restrictive syndromes.^17-19^ Thus, in patients with predominantly right HF and normal or mildly elevated BNP levels, CP should be considered in the differential diagnosis.

Asymptomatic patients should undergo periodical assessment of liver function, functional capacity and jugular venous pressure. The clinical treatment consists in anti-inflammatory drugs, corticosteroids, diuretics and antibiotics. The treatment for tuberculosis should begin prior to surgery and continue after that. Diuretics should be used to reduce, but not eliminate, jugular venous pressure, ascites and edema, because patients need preload to maintain cardiac output. 

Pericardiectomy is the definite treatment for pericardial constriction in symptomatic patients. Increased jugular venous pressure, need for diuretics, evidence of liver failure and reduced functional capacity are indicative of surgery. All our patients were symptomatic and had a long disease course, having thus indication for surgery.

Nine patients (10.7%) died, six in the early postoperative period (within 30 days from surgery), and three, later. Most deaths were due to low cardiac output after pericardiectomy, and two deaths were secondary to cardiogenic and septic shock. In our case series, the following factors were related to death: laboratory markers (anemia, increased inflammatory activity: CRP and BNP levels); clinical marker (AF); and imaging (pulmonary artery hypertension).

The possible mechanism that relates increased mortality and BNP levels has not been totally clarified. We can assume that elevations in that biomarker reflect factors that influence survival, such as myocardial dysfunction, ischemia and increased filling pressures.

Despite the 10.7% mortality, the EuroScore was low. However, the EuroScore study did not contemplate patients undergoing pericardiectomy. We believe, thus, that other perioperative risk scores should be used in patients undergoing pericardiectomy.

Constrictive pericarditis has a variable prognosis with in-hospital mortality ranging from 5% to 16%.^20,21^

Tokuda et al.^22^ have assessed 346 patients submitted to pericardiectomy and found a 10% mortality rate, with the following predictors of worse prognosis: functional class IV; kidney failure; previous cardiac surgery; and use of cardiopulmonary bypass. Peset et al.^21^ have reported a 16% mortality rate, 80% being due to right HF and low cardiac output. Bashi et al.,^23^ however, have reported a reduction in mortality from 16%, in the 1954-73 period, to 11%, in the 1974-86 period, attributed to improvement in postoperative care. In the last period, however, the patients had more advanced disease.

In the study of the Mayo Clinic*,* with 135 patients, there were 39 deaths (29%), 26 of which occurred after hospital discharge. The 5- and 10-year survivals were 71% and 52%, respectively. The survival determinants were: age, previous radiotherapy, functional class, and sodium concentration. Signs and symptoms of functional class II-IV HF were detected in approximately 31% of the patients at some point of their disease course (mean time, 7 months after surgery). The late predictors of HF were age, radiation and ascites.^6^

Similarly to our results, Bertog et al.^20^ have found increased pulmonary artery pressure as an adverse factor, which was attributed to the severity of the concomitant pericardial constriction, myocardial dysfunction or associated pulmonary pathologies.

The ejection fraction can decrease in the postoperative period and can require months to normalize; during that period, the use of digoxin, diuretics and vasodilators can be clinically useful. Other determinants of the prognosis depend on the extent of myocardial atrophy and fibrosis, and on the degree of calcification and adhesion between the epicardium and myocardium, which hinders surgical debridement.

Constrictive pericarditis is curable when early diagnosed and has a good postoperative outcome. Its better understanding and identification will enable us to reduce its high morbidity and mortality rates.

## Conclusions

Constrictive pericarditis manifests with signs and symptoms of biventricular HF, with predominance of right HF, and slightly elevated BNP levels.

The diagnosis is late, with median of 24 months between symptom onset and the correct diagnosis.

Magnetic resonance imaging has better sensitivity for the diagnosis as compared to echocardiography.

Clinical markers (AF), laboratory measures (hemoglobin < 13 g/dL, CRP ≥ 7.4 mg/dL, BNP ≥ 157 pg/mL) and imaging (PASP > 55 mm Hg) were associated with mortality.

### Limitations

The sample consisted mainly of patients with CP of idiopathic etiology, from one single cardiology tertiary center, which can represent bias of selection, and limit the external validity of the results.

Because echocardiography was performed by one single examiner, the reproducibility of the results, that is, intra- and inter-observer variability, could not be assessed. However, the examiner was experienced and blind to the other results.
